# Cardiac Kallikrein-Kinin System Is Upregulated in Chronic Volume Overload and Mediates an Inflammatory Induced Collagen Loss

**DOI:** 10.1371/journal.pone.0040110

**Published:** 2012-06-29

**Authors:** Chih-Chang Wei, Yuanwen Chen, Lindsay C. Powell, Junying Zheng, Ke Shi, Wayne E. Bradley, Pamela C. Powell, Sarfaraz Ahmad, Carlos M. Ferrario, Louis J. Dell’Italia

**Affiliations:** 1 Birmingham Veteran Affairs Medical Center, University of Alabama at Birmingham, Birmingham, Alabama, United States of America; 2 Division of Cardiovascular Disease, Department of Medicine, University of Alabama at Birmingham, Birmingham, Alabama, United States of America; 3 Department of Physiology and Biophysics, University of Alabama at Birmingham, Birmingham, Alabama, United States of America; 4 Xinhua Hospital, Shanghai Jiaotong University School of Medicine, Shanghai, China; 5 Hypertension and Vascular Disease Center, Wake Forest University School of Medicine, Winston-Salem, North Carolina, United States of America; University of Udine, Italy

## Abstract

**Background:**

The clinical problem of a “pure volume overload” as in isolated mitral or aortic regurgitation currently has no documented medical therapy that attenuates collagen loss and the resultant left ventricular (LV) dilatation and failure. Here, we identify a potential mechanism related to upregulation of the kallikrein-kinin system in the volume overload of aortocaval fistula (ACF) in the rat.

**Methodology/Principal Findings:**

LV interstitial fluid (ISF) collection, hemodynamics, and echocardiography were performed in age-matched shams and 4 and 15 wk ACF rats. ACF rats had LV dilatation and a 2-fold increase in LV end-diastolic pressure, along with increases in LV ISF bradykinin, myocardial kallikrein and bradykinin type-2 receptor (BK_2_R) mRNA expression. Mast cell numbers were increased and interstitial collagen was decreased at 4 and 15 wk ACF, despite increases in LV ACE and chymase activities. Treatment with the kallikrein inhibitor aprotinin preserved interstitial collagen, prevented the increase in mast cells, and improved LV systolic function at 4 wk ACF. To establish a cause and effect between ISF bradykinin and mast cell-mediated collagen loss, direct LV interstitial bradykinin infusion *in vivo* for 24 hrs produced a 2-fold increase in mast cell numbers and a 30% decrease in interstitial collagen, which were prevented by BK_2_R antagonist. To further connect myocardial stretch with cellular kallikrein-kinin system upregulation, 24 hrs cyclic stretch of adult cardiomyocytes and fibroblasts produced increased kallikrein, BK_2_R mRNA expressions, bradykinin protein and gelatinase activity, which were all decreased by the kallikrein inhibitor-aprotinin.

**Conclusions/Significance:**

A pure volume overload is associated with upregulation of the kallikrein-kinin system and ISF bradykinin, which mediates mast cell infiltration, extracellular matrix loss, and LV dysfunction–all of which are improved by kallikrein inhibition. The current investigation provides important new insights into future potential medical therapies for the volume overload of aortic and mitral regurgitation.

## Introduction

There is currently no medical therapy that attenuates the eccentric left ventricular (LV) remodeling in the clinical pure volume overload of mitral or aortic regurgitation [1]–[3]. It is of interest that, as opposed to LV pressure overload, LV volume overload is associated with a decrease in interstitial collagen surrounding cardiomyocytes [4]–[6]. The pure volume overload of aortocaval fistula (ACF) in the rat causes a LV stretch stimulus without an increase in LV pressure due to the arterial-venous shunt. As in the human with a pure volume overload, over time this results in an adverse LV eccentric remodeling manifested by increases in LV end-diastolic pressure and LV end-diastolic dimension to wall thickness ratio. We have shown that LV dilatation and collagen degradation occur even before cardiomyocyte elongation [4] supporting the hypothesis that interstitial collagen loss and/or disruption is central to adverse LV remodeling in the volume overload of ACF. We have also shown that the highly tissue specific angiotensin I-converting enzyme (ACE) inhibitor ramipril further exacerbates the loss of collagen and LV eccentric remodeling without improving LV shortening despite decreasing mean arterial pressure [4]. Thus, the antifibrotic and antihypertrophic effects of a pure volume overload are a poor match for renin-angiotensin system blockade and may thus explain why such therapy does not attenuate LV remodeling in patients with isolated MR [2], [3]. Taken together, it is tempting to speculate that ACE inhibition, which decreases angiotensin II and increases bradykinin, exacerbates collagen loss and thus fails to prevent LV dilatation in a pure volume overload.

The potential pathophysioloic role of bradykinin in an isolated volume overload is of particular interest because we have previously demonstrated an increase in interstitial fluid (ISF) of bradykinin [5] and significant attenuation of LV dilatation with bradykinin type 2 receptor (BK_2_) in the early phase of ACF in the rat [4], [5]. In addition to decreasing collagen I and III mRNA production in cardiac fibroblasts [7], bradykinin also modulates matrix metalloproteinase (MMP) activation through the plasminogen activator system [8] and increases MMP-2 and MMP-9 expression [9], [10]. Thus, in the opposite hemodynamic stimulus of pressure overload, kallikrein gene delivery attenuates LV hypertrophy and fibrosis in the spontaneously hypertensive rat [11] and in rats with aortic banding [12]. In addition, direct bradykinin delivery attenuates fibrosis in an experimental model of liver fibrosis [13].

Bradykinin also stimulates proinflammatory cytokine production by mast cells and is chemotactic for polymorphonuclear cells [14], [15]. Mast cells are increased in the acute and chronic phase of ACF [5], [6] and are decreased by treatment with BK_2_ receptor blockade in five day ACF rats [5]. Mast cell degranulation causes the release of proteolytic enzymes like tryptase and chymase, as well as other cytokines that activate MMPs [16]. We have also demonstrated that mast cells possess BK_2_ receptors and that direct bradykinin interstitial infusion causes mast cell degranulation and chymase release into the interstitial fluid space in wild type mice *in* *vivo* [17]. In addition to Ang II-forming capacity, chymase also can activate kallikrein which further increases tissue bradykinin [18].

The purpose of the current study is to understand the mechanisms of extracellular matrix loss in volume overload with the aim of identifying potential new targets that prevent progressive LV remodeling and failure. Through a series of *in vivo* and *in vitro* experiments, we demonstrate that chronic myocardial stretch of a pure volume overload causes upregulation of the kallikrein-kinin system. We present further evidence that the resulting increase in ISF bradykinin in volume overload can mediate mast cell infiltration and extracellular matrix loss that are prevented by kallikrein blockade *in vivo* and result in improved LV function.

## Results

### Morphologic, Hemodynamic and Echocardiographic Data

Total heart weight, LV and RV weight to body weight ratio and lung weight were increased significantly in both 4 and 15 wk ACF vs. age-matched sham rats (Table 1). Mean arterial pressure was decreased >15% at 4 and 15 wk ACF, while LV end-diastolic (ED) pressure and wall stress increased significantly at 4 wk and 15 wk ACF rats (Table 2). However, LV end-systolic (ES) wall stress did not differ from aged-matched shams at 4 and 15 wk ACF due to a decreased in mean arterial pressure that offset the significant increase in LVES dimension. LV +dP/dt_max_ did not differ from age-matched shams at both time points, while LV -dP/dt_max_ at 15 wk ACF was decreased below 15 wk sham rats. LVED dimension (LVEDD), LVED wall stress, and LVEDD/posterior wall thickness ratio were increased at 4 and 15 wk ACF rats (Table 2), consistent with adverse LV remodeling. At 15 wk there was a significant decrease in LV fractional shortening, while at 4 wk ACF LV fractional shortening did not differ from age-matched shams. Although our results showed age-related changes between 4 and 15 wk shams, Bolyut *et al.* has reported that older rats may have a greater cardiovascular susceptibility to isoflurane anesthesia [19]. Nevertheless, directional changes in hemodynamics from sham to ACF at both 4 and 15 wks are the same. Our goal in this study was to investigate time-related responses to ACF volume overload by using age-matched shams.

**Table 1:** Morphologic data at 4 and 15 wk rats.

	4 wk Sham	4 wk ACF	15 wk Sham	15 wk ACF
	(n = 8)	(n = 8)	(n = 10)	(n = 10)
**HW (g)**	0.98±0.05	1.55±0.06*	1.26±0.03^†^	2.23±0.11*
**BW (g)**	359±20	354±9	471±10^†^	489±15
**HW/BW (g/kg)**	2.7±0.1	4.4±0.2*	2.7±0.1	4.8±0.3*
**LVW/BW (g/kg)**	2.1±0.1	3.1±0.1*	1.9±0.1	3.1±0.1*
**RVW/BW (g/kg)**	0.5±0.1	0.9±0.1*	0.5±0.1	1.0±0.1*
**Lung wt (g)**	1.46±0.09	1.84±0.08*	1.97±0.06^†^	2.44±0.11*

Values are expressed as mean±SEM. In each group, values in *parentheses* represent *n*. *P<0.05 vs. age-matched shams. ^†^ *P<*0.05 vs. 4 wk shams. ACF: aortocaval fistula; HW: heart weight; BW: body weight; LVW: left ventricle weight; RVW: right ventricle weight.

**Table 2:** Hemodynamic and echocardiographic data at 4 and 15 wk rats.

	4 wk Sham	4 wk ACF	15 wkSham	15 wkACF
	(n = 8)	(n = 8)	(n = 10)	(n = 10)
**HR (beats/min)**	260±12	271±11	217±12^†^	220±5
**MAP (mmHg)**	114±4	95±3*	95±4^††^	82±4*
**LVEDP (mmHg)**	2±1	7±1*	5±1	9±1*
**LV+dP/dt** **(mmHg/sec)**	8031±440	8947±745	7529±445	6600±289
**LV-dP/dt** **(mmHg/sec)**	−7479±337	−6995±458	−5401±358^†^	−4244±222*
**LVEDD (mm)**	7.0±0.2	10.1±0.3*	9.0±0.1^†^	11.6±0.4*
**LVESD (mm)**	4.3±0.3	6.3±0.3*	5.6±0.1^†^	7.4±0.3*
**LVEDD/PW**	4.8±0.3	7.7±0.5*	5.9±0.3	7.9±0.4*
**LVED-WS (g/cm^2^)**	3±1	14±3**	7±1^†^	16±1*
**LVES-WS (g/cm^2^)**	67±13	77±8	72±7	80±6
**LVFS (%)**	38±4	38±2	40±1	34±2*

Values are expressed as mean±SEM. In each group, values in *parentheses* represent *n*. *P<0.05, **P<0.01 vs. age-matched shams. ^†^ *P<*0.05, ^††^ *P<*0.01 vs. 4 wk shams. ACF: aortocaval fistula; HR: heart rate; MAP: mean arterial pressure; LVEDP: LV end-diastolic pressure; LV+dP/dt: LV pressure derivative maximum; LV-dP/dt: LV pressure derivative minimum. LVEDD: LV end-diastolic dimension; LVESD: LV end-systolic dimension; LVEDD/PW: LV end-diastolic dimension to posterior wall thickness ratio; LVED-WS: LV end-diastolic wall stress; LVES-WS: LV end-systolic wall stress; LV FS: LV fractional shortening.

### Interstitial (ISF) and Plasma Bradykinin, Ang II, Catecholamine

ISF bradykinin levels were increased at 4 and 15 wk ACF vs. age-matched sham rats (Table 3), while ISF Ang II was increased only at 4 wk ACF rats. At 15 wk ACF, ISF Ang II did not differ from age-matched shams, while ISF bradykinin was increased 40% compared to age-matched shams (Table 3). Thus, ISF bradykinin either offset increased ISF Ang II at 4 wk ACF or was increased above ISF Ang II at 15 wk ACF compared to age-matched shams. However, plasma Ang II and bradykinin levels were both increased at 4 and 15 wk ACF rats in response to volume overload. ISF and plasma norepinephrine (NE) and epinephrine (E) did not differ from age-matched shams at 4 wk ACF but were both significantly increased at 15 wk ACF.

**Table 3:** Interstitial and plasma Ang II, bradykinin and catecholamine at 4 and 15 wk rats.

	ISF				Plasma			
	Ang II(pg/hr)	BK(pg/hr)	NE(pg/hr)	E(pg/hr)	Ang II(pg/ml)	BK(pg/ml)	NE(pg/ml)	E(pg/ml)
**4 wk Sham (8)**	1.5±0.2	6.4±0.4	6.2±1.0	3.5±0.3	29±3	10±1	93±12	83±12
**4 wk ACF (9)**	3.0±0.4*	9.3±1.1*	6.8±0.8	5.3±0.9	107±7**	28±2**	89±8	91±21
**15 wk Sham (8)**	1.6±0.2	7.4±1.4	5.9±0.4	3.5±0.4	34±4	17±2	102±16	75±6
**15 wk ACF (10)**	1.9±0.1	12.4±1.7*	10.2±1.0*	8.6±1.1**	65±13*	29±5*	159±17*	101±11*

Values are expressed as mean±SEM. In each group, values in *parentheses* represent *n*. *P<0.05, **P<0.01 vs. age-matched shams. ISF: interstitial fluid; ACF: aortocaval fistula; Ang II: angiotensin II; BK: bradykinin; NE: norepinephrine; E: epinephrine.

### LV ACE, ACE 2, Chymase Activity and Mast Cell Numbers

**Figure 1:**
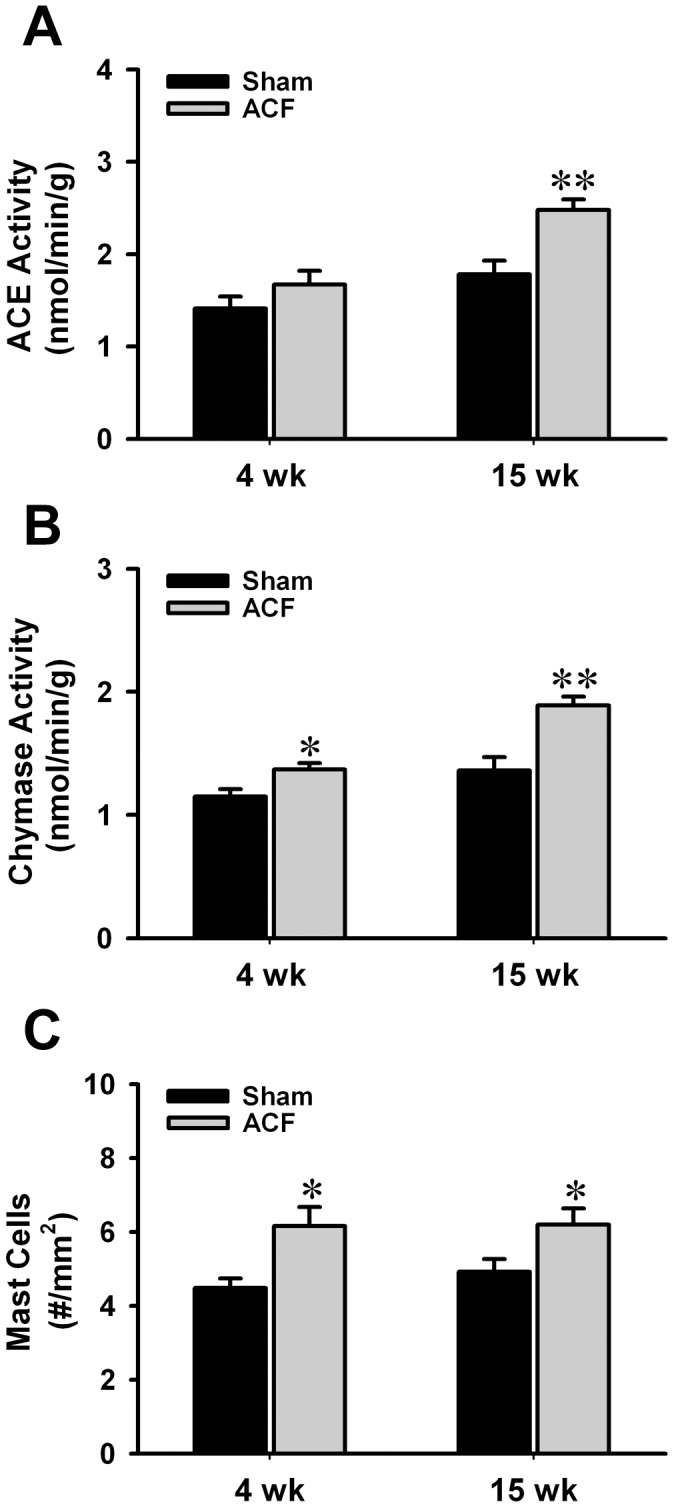
Increasing LV ACE and chymase activity and mast cell numbers in ACF. (A) By using ACE-specific substrate hippuryl-his-leu, LV myocardium ACE activity was defined as captopril-inhibitable hippuric acid (HA) formation expressed as nmoles of HA formed/g/min of tissue. (B) By using Ang I as substrate, chymase-like activity was defined as chymostatin-inhibitable Ang II formation expressed as nmoles of Ang II formed/min/g of tissue. Both activities were measured at 4 and 15 wk age-matched shams and ACF rats. (C) The number of mast cells was quantitatively determined for the entire LV wall using Giemsa-stained paraffin sections. LV mast cell numbers were increased at the 4 and 15 wk ACF rats vs. age-matched shams. Values are mean±SEM. n = 8–10 in each group. *P<0.05, **P<0.01 vs. age-matched shams.

LV ACE activity was increased at 15 wk ACF vs. age-matched sham rats. LV chymase activity and mast cell density increased throughout the course of volume overload (Figure 1, n = 8–10 in each group). Western blots analyses demonstrated unchanged of ACE 2 protein in LV homogenates in age-matched shams (1.08±0.06) and 4 wk ACF rats. However, ACE 2 protein expression was markedly increased at 15 wk ACF rats (1.49±0.12, Figure S1, n = 6). Further, LV ACE 2 activity was increased two-fold at 15 wk ACF vs. age-matched sham rats (11.15±0.30 vs. 5.05±0.52 fmol/min/mg protein, n = 6).

### Kallikrein (KLK) 1, 2, 10, BK_2_R and ACE 2 Expressions in ACF

**Figure 2:**
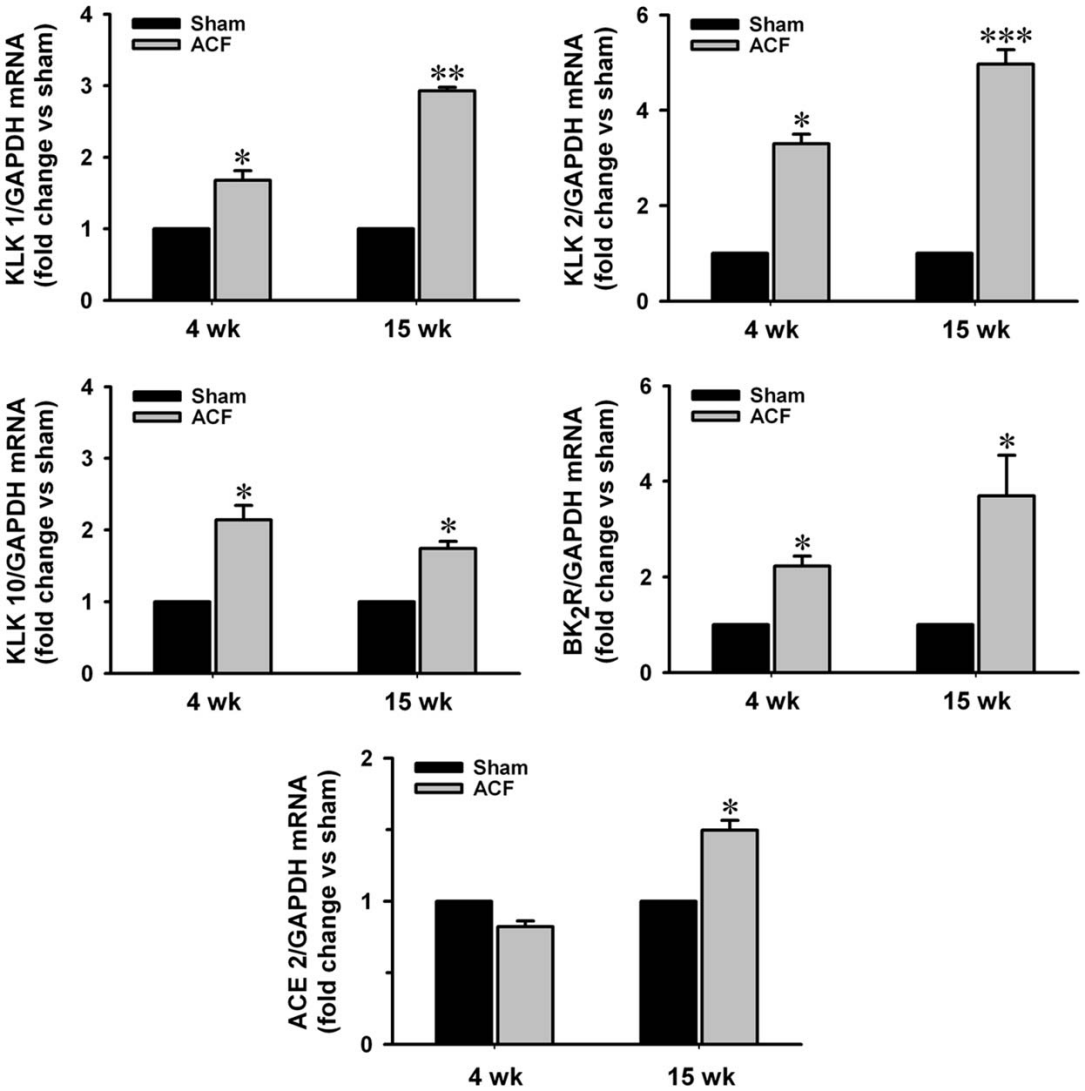
Increasing expressions of kallikreins (KLKs), BK_2_ receptor and ACE 2 mRNA in ACF. Total RNA was extracted from LV tissue with TRIzol reagent. Expressions of kallikrein 1, 2, 10 and BK_2_ receptor and ACE 2 mRNA in normalized to GAPDH at 4 and 15 wk ACF rats compared to age-matched shams. Values are mean±SEM. n = 6–8 in each group. *P<0.05, **P<0.01 and ***P<0.001 vs. age-matched shams.

Kallikrein 1, 2, 10 and BK_2_ receptor mRNA expressions were increased throughout ACF. ACE 2 expression was unchanged at 4 wk ACF rats but increased 50% at 15 wk ACF vs. age-matched sham rats (Figure 2, n = 6–8 in each group).

### Immunohistochemistry of Tissue Kallikrein in LV Myocardium

**Figure 3:**
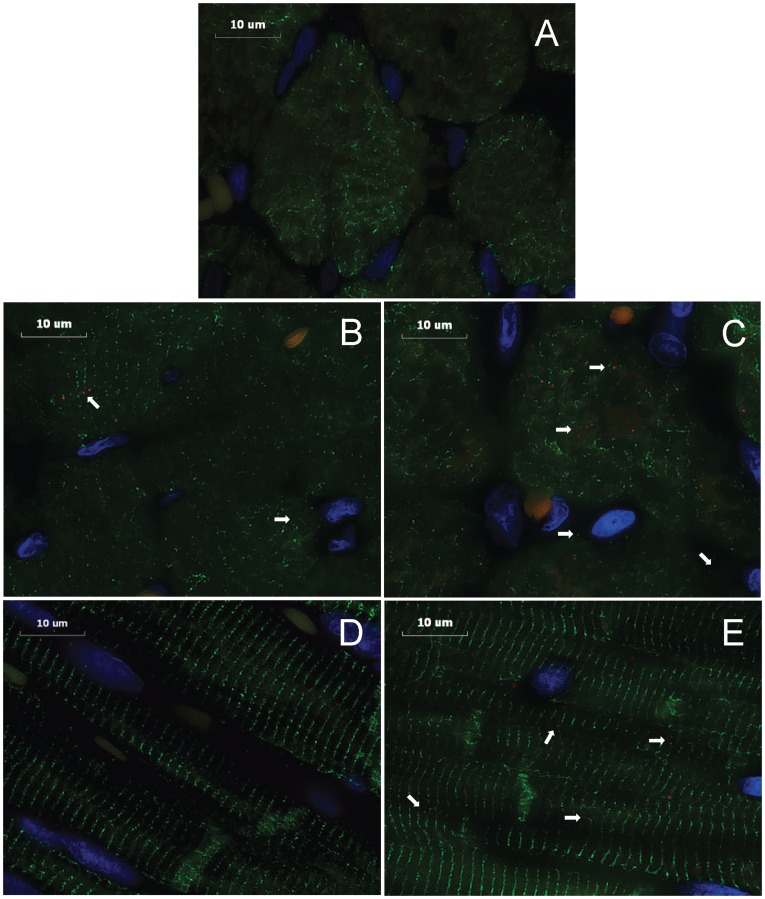
Immunohistochemistry of tissue kallikrein in LV myocardium of sham and ACF rats. Immunohistochemistry were performed on the formalin fixed paraffin-embedded LV. Image acquisition (100x objective, 4000x video-screen magnification) was performed on a Leica DM6000 epifluorescence microscope with SimplePCI software. LV tissues were stained with anti-tissue kallikrein (red), anti-desmin (green), and DAPI (blue). Representative images of the sections: (A) negative control (cross section), (B) 4 wk sham (cross section), (C) 4 wk ACF (cross section), (D) 15 wk sham (longitudinal section), (E) 15 wk ACF (longitudinal section). Arrows demonstrate tissue kallikrein in the LV myocytes or interstitium of each group. Bar scale: 10 µm.

Tissue kallikrein protein expressions were demonstrated in cardiomyocytes and interstitium of the volume overloaded heart (Figure 3). There were increased tissue kallikrein proteins at 4 and 15 wk ACF vs. age-matched sham rats (Figure 3B–E).

### Interstitial Collagen Volume Percent Analysis in ACF

**Figure 4:**
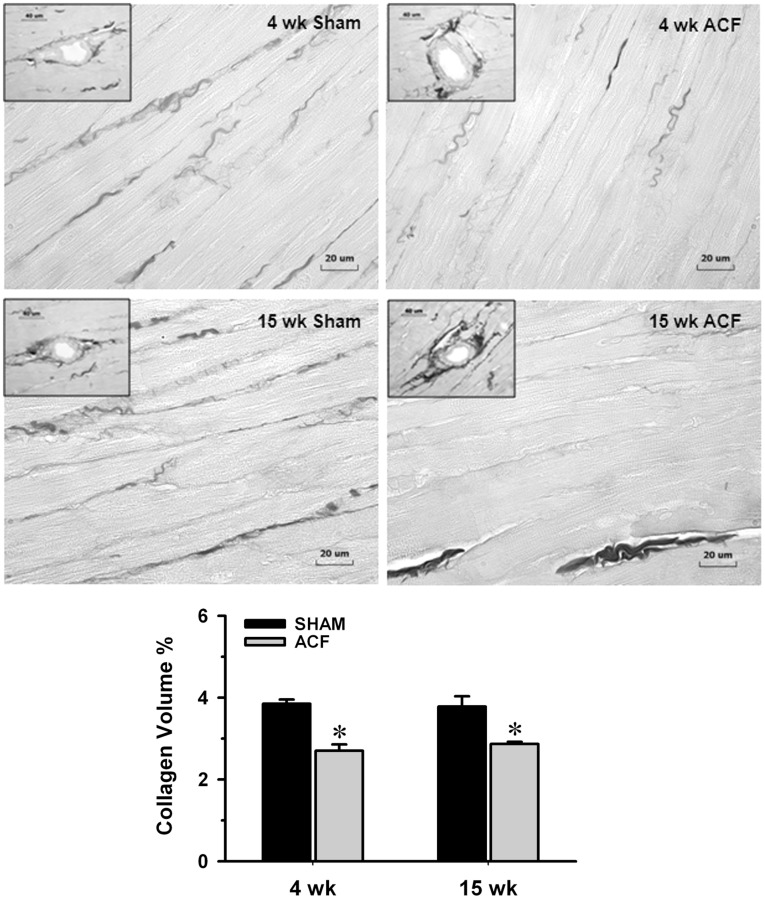
Determination of interstitial collagen volume percent in sham and ACF rats. Representative images of LV interstitial collagen stained with picric acid sirius red F3BA (PASR) were measured at 4 and 15 wk ACF and their age-matched sham rats. The loss of interstitial collagen (shown as dark collagen fibers excluding perivascular areas) between cardiomyocytes at 4 and 15 wk ACF was reflected by the decrease in collagen volume percent (%). However from insets, when comparing the 4 and 15 wk ACF, there is an obvious increase in perivascular collagen compared to sham in the 15 wk ACF. Panel in the bottom displays quantification of the interstitial collagen at 4 and 15 wk ACF and their age-matched shams. Values are mean±SEM. n = 8–10 in each group. *P<0.05 vs. age-matched shams. Bar scale: 20 µm and 40 µm in insets.

Interstitial collagen volume percent by staining with picric acid Sirius red F3BA (PASR) was decreased 30% at 4 and 15 wk ACF vs. age-matched shams (Figure 4, n = 8–10 in each group).

### *In vivo* Effect of Bradykinin on LV Mast Cell Infiltration, Degranulation and Interstitial Collagen Loss

**Figure 5:**
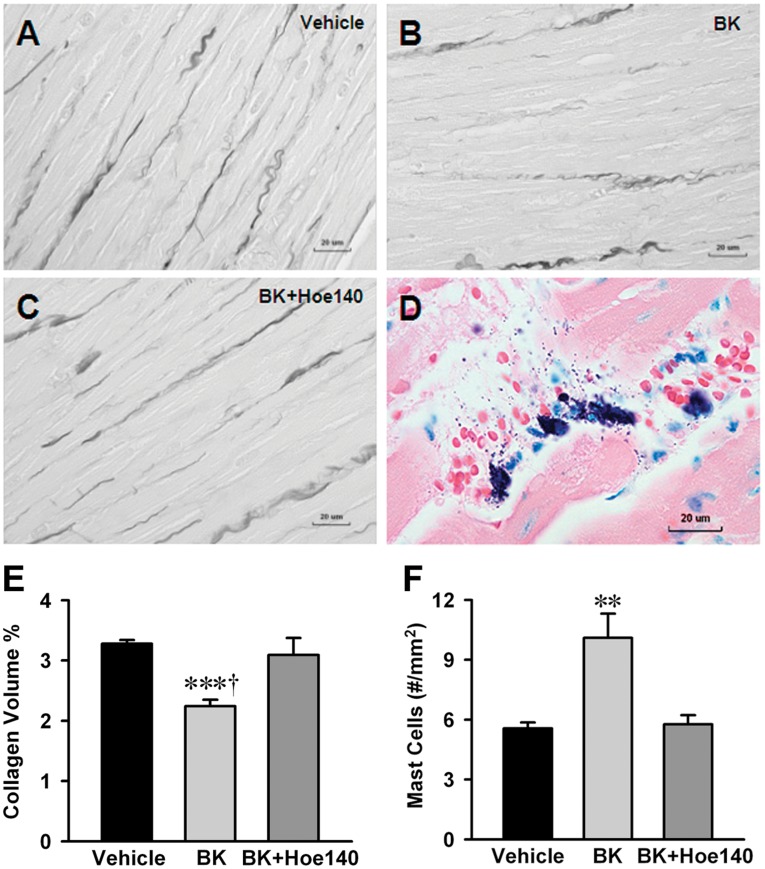
Effect of LV interstitial bradykinin infusion on mast cell infiltration, degranulation and collagen loss. Interstitial collagen volume percent was decreased 30% by bradykinin infusion group (B) compared to saline infused vehicle control (A) and prevented by Hoe-140 (C). Infusion of bradykinin into LV myocardium increased mast cell number by 2-fold compared to vehicle group and mast cell increases were inhibited by Hoe-140 (F). Representative image (D) demonstrates mast cells and mast cell granules show degranulation after interstitial bradykinin infusion. The number of mast cell was quantitatively determined for the entire LV wall using Giemsa-stained paraffin sections. It produces intense staining (purple) specific for mast cell granules. Panel (E) displays quantification of the interstitial collagen of normal rat with interstitial saline or bradykinin (5 ng/ml) infusion for 24 hrs with and without BK_2_R antagonist (Hoe-140, 0.5 mg/kg/d given by osmotic infusion pump). Values are mean±SEM. n = 6 in each group. ***P<0.001 vs. Vehicle. ^†^ *P<*0.05 vs. bradykinin infusion plus Hoe 140. **P<0.01 vs. Vehicle and bradykinin infusion plus Hoe-140. Bar scale: 20 ∝m.

To establish a direct link between ISF bradykinin and extracellular matrix loss, we infused bradykinin (5 ng/ml) into the interstitial space through a microdialysis catheter placed in the LV myocardium of normal rat for 24 hrs with and without BK_2_R antagonist (Hoe-140, 0.5 mg/kg/d given by osmotic infusion pump). Interstitial bradykinin infusion increased LV mast cells two-fold compared to interstitial saline infusion in vehicle control rats, which was prevented by Hoe-140 treatment (Figure 5F, n = 6). In a similar fashion, interstitial collagen volume percent by PASR was decreased 30% in the interstitial bradykinin infusion group and prevented by Hoe-140 compared to vehicle controls (Figure 5A–C and E, n = 6).

### Effect of Aprotinin on Collagen Volume %, Mast Cells, Hemodynamics and LV Remodeling at 2 Day and 4 Wk ACF

**Figure 6:**
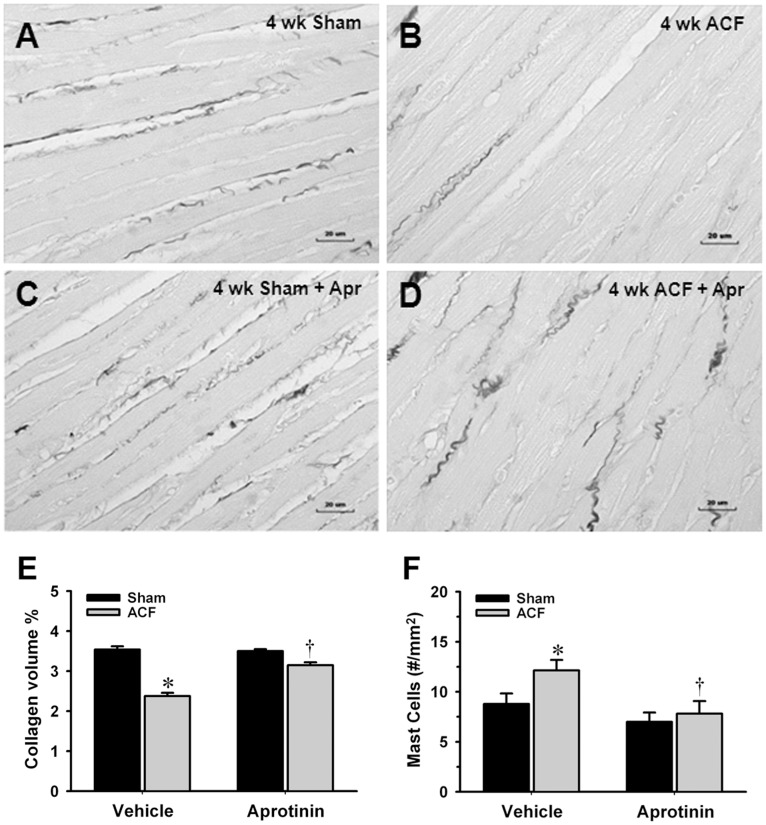
Effect of aprotinin on interstitial collagen volume percent and mast cells in 4 wk ACF. Interstitial collagen volume percent was decreased >30% at 4 wk ACF (B) compared to age-matched sham rats (A) and prevented by aprotinin (D). Panel (C) represents sham with aprotinin treatment which displays no change in the interstitial collagen volume % compared to Vehicle treated sham. Quantification of the interstitial collagen volume % and mast cells were shown in Panels E and F. Mast cell numbers were increased in the 4 wk ACF rats and decreased by aprotinin (F). Values are mean±SEM. n = 7–8 in each group. *P<0.05 vs. Vehicle treated age-matched shams. ^†^ *P<*0.05 vs. Vehicle treated ACF rats.

Aprotinin (12,000 kallikrein inhibitor units, twice daily, sc), a serine protease inhibitor capable of inactivating kallikreins, prevented the decrease in LV interstitial collagen volume percent after 2 day and 4 wk of ACF without an increase in heart rate or mean arterial pressure (Figure 6A–E, n = 7–8, Table 4). LV mast cells were increased >30% at 4 wk ACF compared to age-matched sham rats which were prevented by aprotinin (Figure 6F, n = 7–8). At 2 day ACF, body weight or heart weight/body weight ratio did not differ among sham, ACF, sham+aprotinin and ACF+aprotinin rats (data not shown). Treatment did not attenuate the two-fold increase in LVEDP and had no effect on the increase in LV fractional shortening with ACF. However, at 4 wk post-ACF rats, aprotinin decreased LVES dimension and a trend toward decreased LVES wall stress. This was accompanied by an increase improvement in LV fractional shortening, ejection fraction and a trend of increase velocity of circumferential fiber shortening compared to untreated 4 wk ACF rats.

**Table 4:** Effect of aprotinin on hemodynamics and LV remodeling at 2 day and 4 wk rats.

	2 day				4 wk			
	Sham(n = 5)	ACF(n = 5)	Sham+Apr(n = 5)	ACF+Apr(n = 5)	Sham(n = 7)	ACF(n = 8)	Sham+Apr(n = 7)	ACF+Apr(n = 8)
**HR (beats/min)**	359±6	367±8	371±4	375±12	321±7	307±3	312±5	316±3
**MAP (mmHg)**	100±2	89±3	106±1	84±5*	107±3	90±2*	106±5	86±2*
**LVEDP (mmHg)**	6±1	11±1	5±1	11±2*	4±0	10±1*	4±1	10±1*
**LVESP (mmHg)**	55±3	61±3	58±2	57±4	85±3	75±2	86±5	70±3*
**LV+dP/dt (mmHg/s)**	8767±391	9386±260	8301±442	9065±354	8505±450	8116±348	7931±415	8937±423
**LV-dP/dt (mmHg/s)**	−7474±152	−8797±411	−8474±123	−7879±530	−7379±445	−5948±131*	−7422±236	−6834±333
**LVEDD (mm)**	6.9±0.2	8.2±0.2*	7.0±0.1	8.0±0.1*	7.7±0.2	10.6±0.3*	7.6±0.1	10.2±0.2*
**LVESD (mm)**	4.0±0.2	4.3±0.5	4.1±0.2	4.0±0.2	4.7±0.2	6.8±0.3*	4.7±0.2	5.9±0.2*^†^
**LVEDD/PW**	4.6±0.2	5.8±0.2*	4.6±0.3	5.4±0.4	4.2±0.2	5.7±0.3*	4.5±0.4	6.0±0.4*
**LV FS (%)**	41±2	48±5	43±2	50±3	38±2	37±1	39±1	43±1^†^
**LV EF (%)**	66±3	76±5	72±2	79±3	66±2	63±2	67±2	71±1^†^
**VCFr (%)**	8.3±0.6	10.2±0.5	9.8±0.8	10.1±0.6	7.9±0.8	5.7±0.2	7.5±0.5	7.4±0.6
**LVED-WS (g/cm^2^)**	4±1	16±1*	6±1	16±4*	4±1	15±2*	4±1	16±2*
**LVES-WS (g/cm^2^)**	50±3	54±3	49±9	37±7	65±5	81±8	68±8	62±6
**Coll Vol (%)**	3.9±0.2	2.1±0.1*	4.0±0.3	3.6±0.2^†^	3.5±0.1	2.4±0.1*	3.5±0.1	3.2±0.1^†^

Values are expressed as mean±SEM. In each group, values in *parentheses* represent *n*. *P<0.05 vs. age-matched shams. ^†^ *P<*0.05 vs. ACF. ACF: aortocaval fistula; Apr: aprotinin; HR: heart rate; MAP: mean arterial pressure; LVEDP: LV end-diastolic pressure; LV+dP/dt: LV pressure derivative maximum; LV-dP/dt: LV pressure derivative minimum. LVEDD: LV end-diastolic dimension; LVESD: LV end-systolic dimension; LVEDD/PW: LV end-diastolic dimension to posterior wall thickness ratio; FS: fractional shortening; EF: ejection fraction; VCFr: velocity of circumferential fiber shortening; LVED-WS: LV end-diastolic wall stress; LVES-WS: LV end-systolic wall stress; Coll Vol (%): interstitial collagen volume percent.

### *In vitro* Effect of Cell Stretch on Kallikrein Kinin and Gelatinase Expression

Primary cultured adult cardiac myocytes and fibroblasts underwent cyclic mechanical stretch to simulate volume overload. Cyclic stretch of these cells produced a >two-fold increase kallikrein 1, 10 and BK_2_R mRNA expressions in cell lysate and prevented by aprotinin (Figure S2, n = 4–5 in each group). In cardiomyocytes, bradykinin level increased 6-fold in the culture media (1.26 vs. 7.83 ng/ml, un-stretched vs. 24 hrs stretched, *P<*0.01, n = 6). In the fibroblast culture media, bradykinin levels increased from undetectable value (un-stretched) to 1.64 ng/ml after 24 hrs stretched (n = 6). Cyclic stretch of adult rat cardiomyocytes and fibroblasts produced a little increase in MMP-9 and two-fold increases in MMP-2 activity (Figure S3, n = 4–5), respectively. Western blots showed undetectable MMP-9 expression but a >30% increase MMP-2 expression in both stretched cells and inhibited by aprotinin (Figure S3, n = 4–5).

## Discussion

In the current investigation, we show how the increase in the LV kallikrein-kinin system expression can explain interstitial collagen loss and inflammatory cell infiltration that have been central to the pathophysiology of LV dilatation in a chronic pure volume overload. Rat cardiomyocytes produce mRNA for kininogen, kallikrein, and kinin receptors [20] and the rat heart contains a kininogen-like protein that is elevated with acute inflammation [21] and chronic volume overload [22]. To connect kallikrein-kinin system upregulation with the myocardial stretch of volume overload, the current study demonstrates that 24 hrs cyclic stretch of adult cardiac myocytes and fibroblasts results in increased cellular kallikreins and BK_2_R mRNA expression and increased bradykinin release. Further, kallikrein protein is demonstrated in cardiomyocytes and LV ISF bradykinin is increased in ACF *in vivo*, supporting cardiomyocyte and fibroblast bradykinin production with volume overload.

The loss of collagen and increased kallikrein-kinin system expression with ACF contrasts with an increase in collagen synthesis and down regulation of LV BK_2_ receptor mRNA in the pressure overloaded rat heart [23]. Thus, it is tempting to speculate that upregulation of the LV kallikrein-kinin system in volume overload produces an anti-fibrotic phenotype that promotes a more compliant LV chamber. However, the deleterious effects of prolonged kallikrein-kinin system expression and collagen loss in volume overload pose a unique therapeutic problem, because treatment with conventional renin-angiotensin system blockade decreases rather than promotes collagen production. In the current study, kallikrein inhibitor-aprotinin prevents loss of interstitial collagen in 2 day and 4 wk of ACF without an increase in mean arterial pressure. Aprotinin also decreases LVES dimension and increases in fractional shortening and ejection fraction, along with trends toward increased velocity of circumferential shortening and +dP/dt_max_ and –dP/dt_max_. We speculate that improvement in these load dependent indices of LV function results from a more efficient transmission of forces between adjacent myofibers and myocytes due to preservation of interstitial collagen.

We postulate that the acute (2 day) and subacute (4 wk) volume overload causes upregulation of kallikrein and bradykinin resulting in a negative extracellular matrix homeostatic balance in which MMP activation dominates over collagen synthesis. Dolgilevich *et* *al.* demonstrate a decrease in collagen mRNA at 45 days and an increase in collagenase mRNA expression at 14 days of ACF [24]. Further, MMP-2 activity is increased in both acute and chronic ACF [6], [25]. Stretch of cardiac fibroblasts *in vitro* has also been shown to increase collagenase activity and membrane type-1 MMP expression [26]. We also show that cyclic stretch of adult cardiomyocytes and fibroblasts produces a 20% increase in MMP-9 and two-fold increase MMP-2 gelatinase activity and kallikrein 1 gene expression that are decreased by aprotinin (Figures S2 and S3). These results *in vitro* support the beneficial effect of aprotinin in preserving interstitial collagen and improving LV function in the 2 and 4 wk ACF.

Another important component to the pathophysiology of LV remodeling in a pure volume overload is inflammatory cell infiltration [25], [27], in particular mast cells in the dog model of isolated mitral regurgitation [28] and in the rat with ACF [5], [6]. Bradykinin is a well known chemotactic stimulus for many types of inflammatory cells [29]. We have demonstrated BK_2_ receptors on mast cells and that BK_2_ receptor blockade prevents ACE inhibitor-mediated increases in LV ISF chymase activity in the normal mouse [17] and mast cell accumulation in the 5 day ACF rat [5]. To connect ISF bradykinin with mast cell and collagen loss, the current study demonstrates that cardiac interstitial bradykinin infusion increases mast cell numbers and causes collagen loss, both of which are prevented by BK_2_ receptor blockade. Furthermore, kallikrein inhibitor-aprotinin prevents the increase of mast cell numbers and loss of interstitial collagen in 4 wk ACF.

It is important to note that in the 15 week ACF there are patchy areas of LV endocardial perivascular fibrosis, despite the loss of collagen surrounding cardiomyocytes. A similar finding has been reported in the failing human heart where perivascular fibrosis occurs in combination with loss of cardiomyocyte interstitial collagen [30]. Taken together, these findings suggest a compartmentalization of collagen homeostasis in the failing heart, with collagen loss surrounding cardiomyocytes and a perivascular fibrosis. However, it is of interest that in the aortas of 30 month old rats, MMP pro-inflammatory effects result in an increase in collagen formation and vascular stiffening [31]. It is important to note that in the current investigation, ACF is induced at 10 week old rats and continues for 15 weeks–raising the question whether a more diffuse pattern of fibrosis may occur in older rats.

The current study also reports discordant ISF and plasma Ang II levels. In the rat, the largely interstitial enzyme chymase has multiple isoforms, in particular β-chymase, which can degrade ISF Ang II [32]. In addition, ACE 2 mRNA and protein are increased along with a two-fold increase in ACE2 activity in the LV of 15 wk ACF (Figure S1). ACE 2 is a carboxypeptidase that converts Ang I and Ang II to Ang-(1–7) [33], [34]. Ang-(1–7) exerts counter-regulatory effects of Ang II through a specific pathway involving a novel Ang-(1–7) receptor and is an endogenous inhibitor of the C-terminal active site of ACE [33]. More importantly, ACE 2 has a catalytic activity for Ang II that is 400 fold higher than for Ang I. Taken together, the decrease ISF Ang II in the 15 wk ACF may be due to an increased β-chymase activity and ACE 2 activity, resulting in a decrease ISF Ang II and increase ISF Ang-(1–7), which in combination with the increase in ISF BK decrease interstitial collagen production in the 15 wk ACF.

**Figure 7:**
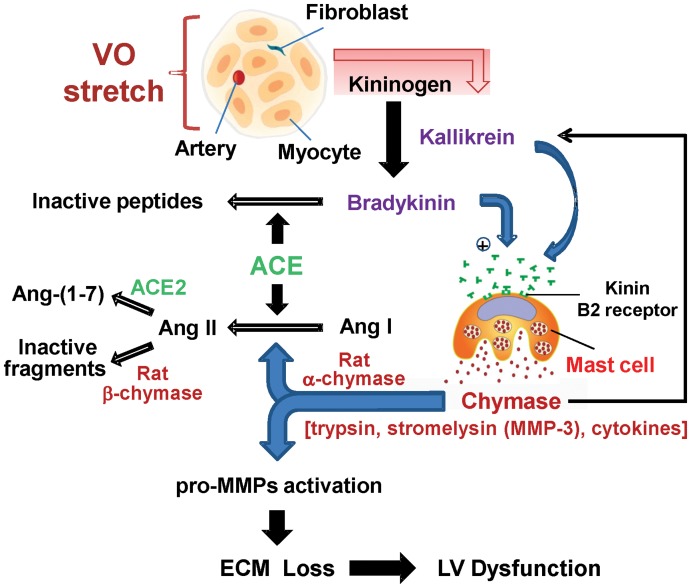
A diagram demonstrates the hypothesized pathway of volume overload stress effect on LV remodeling and functions. The pure stretch of volume overload induces cardiac kallikrein-kinin upregulation from cardiomyocytes and fibroblasts that result in increased ISF bradykinin and activate kinin B2 receptor. Kallikrein and kinin cause mast cell degranulation. Mast cell degranulation causes the release of proteolytic enzymes like tryptase and chymase, as well as other cytokines that activate MMPs. Chymase also can activate kallikreins, MMPs and degrade collagen and non-collagen extracellular matrix components, such as fibronectin. The increase in bradykinin can further decrease extracellular matrix synthesis. Furthermore, tissue kallikreins can activate the kinin B_2_ receptor in the absence of kininogen. Because the kininogen substrate is abundant in plasma and tissues, the expression and availability of tissue kallikrein are the rate-limiting factors in kinin production.

The *in vivo* and *in vitro* results of the current investigation summarized in Figure 7 demonstrate that a pure volume overload/stretch is associated with upregulation of the kallikrein-kinin system and increased ISF bradykinin, which mediates mast cell infiltration, extracellular matrix loss, and LV dysfunction–all of which are improved by kallikrein inhibition-aprotinin. However, at 15 wk ACF stage, there is an increase in LV ISF catecholamines, perivascular fibrosis, and a decrease in LV function, which at that time point forward may benefit from full rennin-angiotensin system blockade. However, in the early phase, ACE inhibition may in fact exacerbate the process by causing a further increase in ISF bradykinin, a decrease extracellular matrix synthesis, and mast cell recruitment and degranulation. This study demonstrates that LV remodeling is truly a diverse process that is dependent upon the type and stage of the cardiac stress, as well as the age of the patient. In the particular case of a pure volume overload, studies are ongoing to uncover novel drug targets that control inflammation and collagen loss in the early stages and thus attenuate LV remodeling and prolong the time to development of heart failure.

## Methods

### Ethics Statement

These studies conform to the Guide for the Care and Use of Laboratory Animals published by the US National Institutes of Health (NIH Publication No. 85–23, revised 1996), and was approved by the University of Alabama at Birmingham’s Institutional Animal Care and Use Committee (Approved protocol #111009251). The protocol for purification of tissue kallikrein antibody was approved by the Institutional Animal Care and Use Committee at the Medical University of South Carolina.

### Creation of Aortocaval Fistula (ACF) in the Rat

ACF or sham surgery was performed on 10-week old male Sprague-Dawley rats (200–250 g) under sterile conditions using isoflurane anesthesia as previously described in our laboratory [4], [5]. Briefly, a ventral abdominal laparotomy was performed to expose the aorta and caudal vena cava ∼1.5 cm below the renal arteries. Blunt dissection removed the overlying adventitia and exposed the vessels, taking care not to disrupt the tissue connecting the vessels. Both vessels were then occluded proximal and distal to the intended puncture site, and an 18-guage needle was inserted into the exposed abdominal aorta and advanced through the medial wall into the vena cava to create the fistula. The needle was withdrawn and the ventral aortic puncture was sealed with cyanoacrylate. Creation of the ACF was visualized by the pulsatile flow of oxygenated blood into the vena cava. The abdominal musculature and skin incisions were closed by standard techniques with absorbable suture and autoclips. The control animals, sham, underwent general anesthesia and an abdominal incision without ACF.

### Cardiac Microdialysis *in vivo* and Interstitial Fluid Collection

Cardiac microdialysis probes were inserted into the LV myocardium in 4 and 15 wk ACF and in age-matched sham rats. Interstitial fluid collections were performed in the conscious state as previously described in our laboratory [5]. Each microdialysis probe had a semipermeable membrane fiber (AN69, Hospal, Lyon, France) with a molecular mass cutoff of 35 kDa (internal diameter ≈250 µm) with Pebax tubing (outer diameter ≈200 µm) inserted and sealed in each end of the fiber such that ∼4 mm of fiber remained exposed at the center of the assembly; this exposed region resided within the LV myocardium free wall. After implantation, the probe was perfused with 0.9% saline using a precision infusion syringe pump (BAS, IN) at a flow rate of 1.0 µl/min. The dialysate was collected from the outflow tubing in eppendorf tubes on ice with 5 µl of EGTA with reduced glutathione for catecholamine, 10 µl acetic acid (1.0 M) for Ang II peptides, and 98% ethanol for BK and frozen at −80°C until biochemical analysis. Two 10-hr dialysate were collected for ISF bradykinin and Ang II and two 4-hr dialysate were collected for norepinephrine (NE) and epinephrine (E) analysis over a total 28 hr period beginning 24 hrs after surgery recovery from dialysis probe implantation. At the end of the interstitial collection, Evans blue was introduced into the probe to visually check if the probe was positioned correctly in the LV.

### Osmotic Pump Infusion of BK

We infused BK (5 ng/ml) into the interstitial space through a microdialysis catheter placed in the LV myocardium of normal rat for 24 hours with and without BK_2_R antagonist (Hoe-140, 0.5 mg/kg/day, American Peptide Company, Sunnyvale, CA) and was given by osmotic infusion pump subcutaneous (model 2001, 7 days, Alzet, Cupertino, CA,).

### Hemodynamic and Echocardiographic Measurement

Because of the potential effects of the probes and infusions on tissue, we did not use above microdialysis rats for tissue analysis. A subset of animals was used for hemodynamic and echocardiographic measurement. At the time of sacrifice aortic pressure, LV peak systolic and LV end-diastolic pressure, peak +/− dP/dt were measured in anesthetized animals with an intravascular pressure transducer (model SPR-249A Millar Mikro-Tip catheter transducer, Millar Instruments, Houston, TX) introduced via the right carotid artery into the LV chamber. High-fidelity LV pressure was recorded concurrent with echocardiography (Agilent Sonos 5500, Philips, Bothel, WA). LV wall stress and function were calculated as previously described [4]. Venous blood was collected for BK and Ang II measurement under isoflurane anesthesia by venous puncture of the abdominal inferior vena cava. After sacrifice, whole heart, LV, RV and lungs were weighed. LV tissue was collected for evaluation of kallikreins, BK_2_ receptor and ACE 2 expressions, chymase activity, mast cell numbers and interstitial collagen volume percent.

### Ang II, BK and Catecholamine Concentration

Plasma Ang II concentration was determined by a method described from our laboratory that combined HPLC and radioimmunoassay (RIA). ISF Ang II samples were determined by direct RIA; and plasma, ISF bradykinin concentrations were determined using a standard RIA kit (Phoenix Pham., Mountain View, CA) [5]. Plasma, ISF NE and E concentrations were determined using a standard ELISA kit (Immuno-Biological Lab., Minneapolis, MN).

### LV Myocardial ACE, ACE 2 and Chymase-like Activity

By using ACE-specific substrate hippuryl-his-leu, ACE activity was defined as captopril-inhibitable hippuric acid (HA) formation expressed as nmoles of HA formed/g/min of tissue. By using Ang I as substrate, chymase-like activity was defined as chymostatin-inhibitable Ang II formation expressed as nmoles of Ang II formed/min/g of tissue [5]. ACE 2 activity was calculated based on adding 1 nmol/L of ^125^I-Ang-(1–12) substrate to the reaction mixture and determining the amount of Ang II product formation. The enzyme activity was defined as fmoles of Ang II product formation from ^125^I-Ang-(1–12) substrate per min per mg of protein (fmol Ang II formation/min/mg protein) [35].

### Western Blot Analysis

Western blots analyses for ACE 2 (rabbit antibody, 1∶1,000), MMP-2 (rabbit antibody, 1∶1,000), and MMP-9 (rabbit antibody, 1∶500) were performed, and bands were quantified as previously described by our laboratory [24]. β-actin (mouse antibody 1∶1,000) was used as an internal control. All antibodies were purchased from Abcam, Cambridge, MA. An equal amount of protein (20–30 µg) sample was loaded and the optical density of bands was quantified by Image J 1.44p (NIH image).

### Gelatin Gel Zymography

Cell lysates (3–10 µg of protein) were loaded in a precast 10% gelatin gel, and gel zymography was performed following the manufacturer’s instruction (Novex, Invitrogen) as previously described by our laboratory [24]. A proteolytic bands of 78 kDa (MMP-9) and 62 kDa (MMP-2) were scanned and quantified with Image J. Data are expressed as fold changes of the corresponding control.

### Real-time RT-PCR

Total RNA was extracted from LV with TRIzol reagent (Invitrogen Corporation, Carlsbad, CA). Messenger RNA was reversed transcribed using Transcriptor Reverse Transcriptase (Roche Diagnostics Corporation, Indianapolis, IN). Real-time PCR reactions were carried out using LightCycler and LightCycler-FastStart DNA Master SYBR Green I kit (Roche Diagnostics Corporation). The primers set for ACE 2, Kallikreins 1, 2, 10, BK_2_R and GAPDH were as follows: for ACE 2, 5′ primer was 5′-GTCCAAGATCGCCCAAAATTTC and 3′ primer was 5′-TTTCTACGTCTTCGATCAACT (454 bp); for kallikrein 1, 5′ primer was 5′-AAAGCCCACACACAGATGGT and 3′ primer was 5′-TAGCTGGCCTATTGGTTTCG (169 bp); for Kallikrein 2, 5′ primer 5′-GCTGTCATCAATGAATACCTC and 3′ primer was 5′-TGGTCATGCACAGGTTGTTCA (224 bp); for kallikrein 10, 5′ primer 5′-GCACCAAACCCCTGAATTGGG and 3′ primer was 5′-ACACCTGGGTTATAGGGTTCA (250 bp); for BK_2_R, 5′ primer was 5′-CCATCTCTCCACCTGCATTG and 3′ primer was 5′-CGTCTGGACCTCCTTGAACT (739 bp); and for GAPDH, 5′ primer was 5′-ATGGTGAAGGTCGGTGTG and 3′ primer was 5′-ACCAGTGGATGCAGGGAT (633 bp). Real-time PCR conditions for the primers were optimized at 3 mM Mg^2+^, 0.5 µM primers and 40 ng cDNA in a total reaction volume of 20 µl. All PCR products appeared as single bands of expected molecular size by 1.5% agarose gel fractionation; they were cloned and sequenced to identify the target cDNAs. We also verified that each PCR product consisted of only a single species, as determined by melting curve analysis. To determine copy number, standard curves for each target transcript were established using purified PCR products. They were linear and correlation coefficients were greater than 0.99.

### Quantitative Evaluation of Myocardial Mast Cells

The number of mast cells was quantitatively determined for the entire LV wall using Giemsa-stained paraffin sections. This method produces intense staining (purple) specific for mast cell granules. 50–70 fields were examined using the 20x objective of the microscope, and the number of mast cells per mm^2^ was tabulated as previously described [4], [5].

### Interstitial Collagen (Collagen Volume Percent) Analysis

Hearts were removed from each rat at sacrifice and immersion-fixed in 10% buffered-formalin. Horizontal short-axis sections through the mid-LV were embedded in paraffin, sectioned at 3 µm thickness, and stained with picric acid sirius red F3BA (PASR). Quantitative analysis of interstitial collagen was accomplished by light microscopy with a video-based image-analyzer system. Collagen volume percent was quantitatively evaluated at high power (40x objective, 1550x video-screen magnification) with a 540-nm green filter to provide grayscale contrast of dark collagen fibers with light background. Using images collected by digital camera, we determined the collagen volume percent of 30–40 randomly selected fields in each section, excluding perivascular areas, and the mean value was calculated for each animal. All measurements were performed in a blinded manner.

### Immunohistochemistry for Tissue Kallikrein in LV Myocardium

Immunohistochemistry were performed on the formalin fixed paraffin-embedded LV from rats. 5 µm sections were mounted on slides, deparaffinized in xylene and rehydrated in a graded series of ethanol. After blocking with 1x PBS/1% Casein, overnight incubation at 4°C with tissue kallikrein antibody (1∶1000; provided by Dr. Julie Chao; Medical University of South Carolina, Charleston, South Carolina, United States of America), followed by 1 hr incubation room temperature with desmin antibody (Abcam, Cambridge, MA, 1∶200) was performed. Alexa Fluor 594- and Alexa Fluor 488-conjugated secondary antibodies (Molecular Probes, Eugene, OR; 1∶500 each) was applied to visualize the tissue kallikrein (red) and desmin (green) in the tissue. Nuclei were stained (blue) with DAPI (1.5 µg/ml; Vector Laboratories, Burlingame, CA). Image acquisition (100x objective, 4000x video-screen magnification) was performed on a Leica DM6000 epifluorescence microscope with SimplePCI software (Compix, Inc., Cranberry Township, PA). Images were adjusted appropriately to remove background fluorescence.

### Cardiac Myocytes and Fibroblasts Isolation

Cardiomyocytes and fibroblasts were isolated from the 10-wk-old Sprague-Dawley rat heart. The heart was perfused with perfusion buffer (120 mmol/L NaCl, 15 mmol/L KCl, 0.5 mmol/L KH_2_PO_4_, 5 mmol/L NaHCO_3_, 10 mmol/L HEPES, and 5 mmol/L glucose, at pH 7.0) for 8 min and digested with perfusion buffer containing 2% collagenase II for 25 min at 37°C. The right ventricle and atria were removed before the perfused-heart was minced. The cell suspension was then mixed with stop buffer containing 10% fetal bovine serum (FBS). After filtered, the flow-through was spun at 300 rpm for 3 min to pelletize the cardiomyocytes. After calcium re-introduction, only cardiomyocytes with purity and viability (rod-shaped) >95% or 80%, respectively, were used.

The supernatant was then resuspended in DMEM supplemented with antibiotics (penicillin/streptomycin, 1%), L-glutamine ascorbate and 10% FBS. Cells were subjected to differential plating on uncoated cell culture plates (100 mm^2^) for 45 minutes. Non-adherent cells (mostly cardiomyocytes and smooth muscle cells) were removed and fresh medium added. Cells were grown to 95% confluence and then detached by 0.05% trypsin/EDTA and plated on the Bioflex culture plates with precoated collagen I. Cultured cells were immunophenotyped by their absence of alpha smooth muscle actin which was expressed in myofibroblast, but presence of vimentin as fibroblast. From the staining passage one of the fibroblasts with alpha smooth muscle actin (Figure S4, A, green), vimentin (Figure S4, B, red) and DAPI (nuclei, blue), we showed >95% purity (Figure S4, C, composite) of the prep and little myofibroblast differentiation. Cardiomyocyte and fibroblast used in the passage one were studied after deprived of serum for 24 hrs prior to cyclic stretch study.

### Application of Extrinsic Mechanical Load

Cells (2x10^6^) were cultured on laminin-coated Flexcell plates (Flexcell International Corp., Hillsborough, North Carolina, United States of America) in DMEM medium containing 10% FBS, 2 mM glutamine, 10 U/mL penicillin, and 100 mg/mL streptomycin. The medium without serum was changed 24 hrs before initiation of the experiment. Cultured rat ventricular myocytes or fibroblasts with or without aprotinin (12,000 kallikrein inhibitor units in the medium) were subjected to cyclic strain (60 cycles/min) on the Flexcell Strain apparatus (model FX-5000; Flexcell International, Hillsborough, North Carolina, United States of America) at a level of distension sufficient to promote an increment of approximately 5% in surface area at the point of maximal distension on the culture surface. The cyclic stretch will be performed for 24 hrs and without interruptions. Control cells were grown in identical culture plates and incubated in the same incubator as the stretched cultures, but were not mounted in the Flexercell Strain Unit.

### Statistical Analysis

All data were presented as mean±standard error of mean (SEM). An unpaired Student’s t-test was used to compare 4 wk vs. 15 wk sham groups, and to compare age-matched shams vs. ACF groups. To test the efficacy of aprotinin in ACF, a one-way analysis of variance (ANOVA) combined with multiple comparisons was performed using the Holm-Sidak method in Table 4. A p value of less than 0.05 was required for statistical significance.

## Supporting Information

Figure S1 **ACE 2 protein expression in the LV at 4 and 15 wk ACF and age-matched sham rats.** Total protein was extracted from LV tissue and ACE 2 protein expression was normalized by β-actin. Representative image of western blots of LV extracted from sham and ACF rats (upper panels). Quantification of the bands is shown at lower panels. Values are mean±SEM. n = 6–8 in each group. **P<0.01 vs. age-matched shams.
(TIF)

Figure S2 **Expressions of kallikreins (KLKs) and BK_2_ receptor in stretched cells.** Expressions of kallikrein (KLK) 1, 10 and BK_2_R mRNA in normalized to GAPDH in response to cyclic stretch at 5% maximum strain and 1 Hz for 24 hrs with or without aprotinin in adult cardiac myocytes and fibroblasts. Vehicle unstretched cells were grown in identical culture plates and incubated in the same incubator as the stretched cultures, but were not mounted in the Flexercell Strain Unit. Values are expressed as mean±SEM. n = 4–6 in each group. *P<0.05, **P<0.01 vs. Vehicle.
(TIF)

Figure S3 **Gelatinase activity and protein expression in stretched cells.** Gel zymography of gelatinase activity and MMP-2 protein expression in 24 hrs of cyclic stretch with or without aprotinin in adult rat cardiac myocytes and fibroblasts. There is increased MMP-9 activity at 78 kDa in cardiomyocytes (Panel A) and MMP-2 activity at 62 kDa in fibroblasts (Panel B) after 24 hrs of stretch. Panels C and D, MMP-2 protein expressions were increased in response to stretch in both cells. The gelatinase activity and MMP-2 protein expressions were both reduced by aprotinin treatment. Values are expressed as mean±SEM. n = 4–6 in each group. *P<0.05 vs. Vehicle.
(TIF)

Figure S4 **Immunofluorescence revealed phenotype of cardiac fibroblast.** Cardiac fibroblasts used in our studies were at passage one. From the staining of the fibroblasts with alpha smooth muscle actin (A, green), vimentin (B, red) and DAPI (nuclei, blue), we showed >95% purity (C, composite) of the prep and little myofibroblast differentiation.
(TIF)
