# Supplementary figures and images for: Cardiac Kallikrein-Kinin System Is Upregulated in Chronic Volume Overload and Mediates an Inflammatory Induced Collagen Loss
Source: PLoS One. 2012 Jun 29;7(6):e40110. doi: 10.1371/journal.pone.0040110 (PMC3387019; doi:10.1371/journal.pone.0040110)

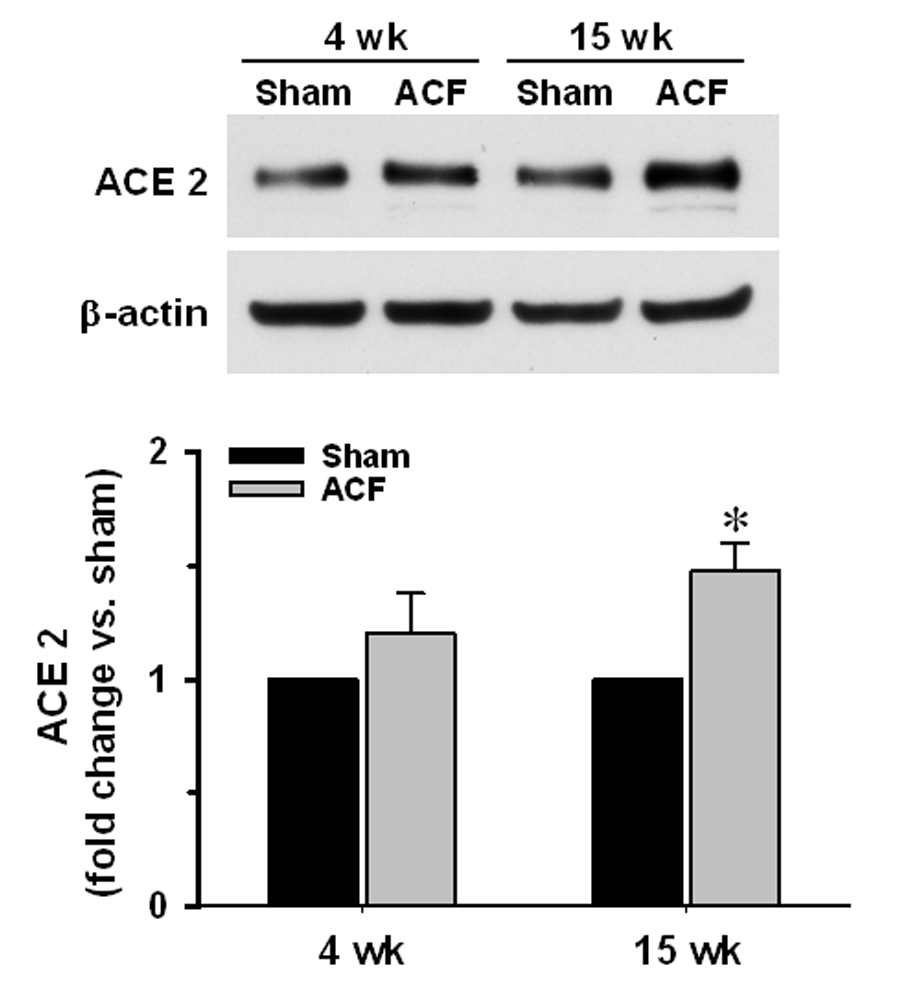

Supplement: Figure S1 — ACE 2 protein expression in the LV at 4 and 15 wk ACF and age-matched sham rats. Total protein was extracted from LV tissue and ACE 2 protein expression was normalized by β-actin. Representative image of western blots of LV extracted from sham and ACF rats (upper panels). Quantification of the bands is shown at lower panels. Values are mean±SEM. n = 6–8 in each group. **P<0.01 vs. age-matched shams. (TIF) [file pone.0040110.s001.tif]

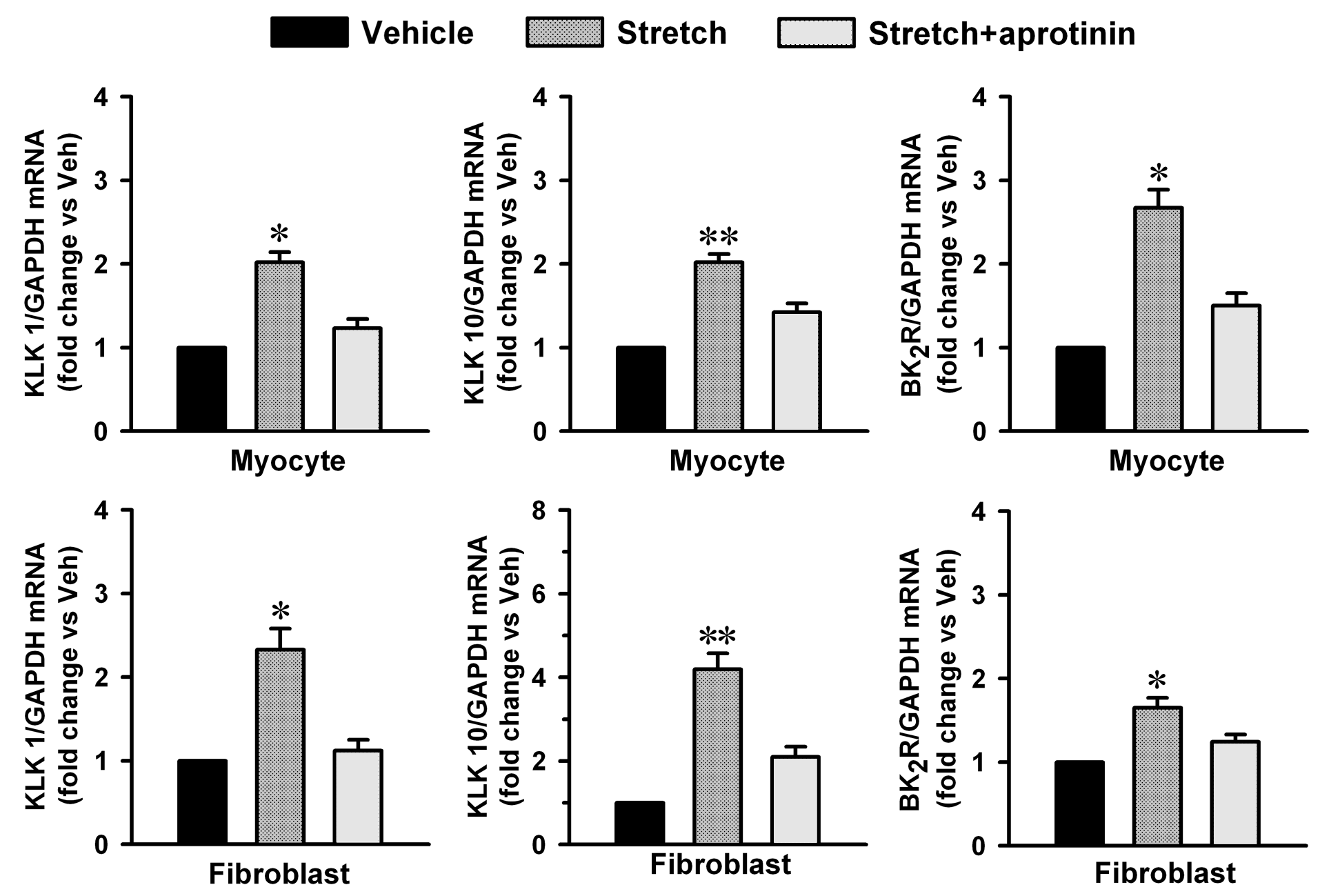

Supplement: Figure S2 — Expressions of kallikreins (KLKs) and BK2 receptor in stretched cells. Expressions of kallikrein (KLK) 1, 10 and BK2R mRNA in normalized to GAPDH in response to cyclic stretch at 5% maximum strain and 1 Hz for 24 hrs with or without aprotinin in adult cardiac myocytes and fibroblasts. Vehicle unstretched cells were grown in identical culture plates and incubated in the same incubator as the stretched cultures, but were not mounted in the Flexercell Strain Unit. Values are expressed as mean±SEM. n = 4–6 in each group. *P<0.05, **P<0.01 vs. Vehicle. (TIF) [file pone.0040110.s002.tif]

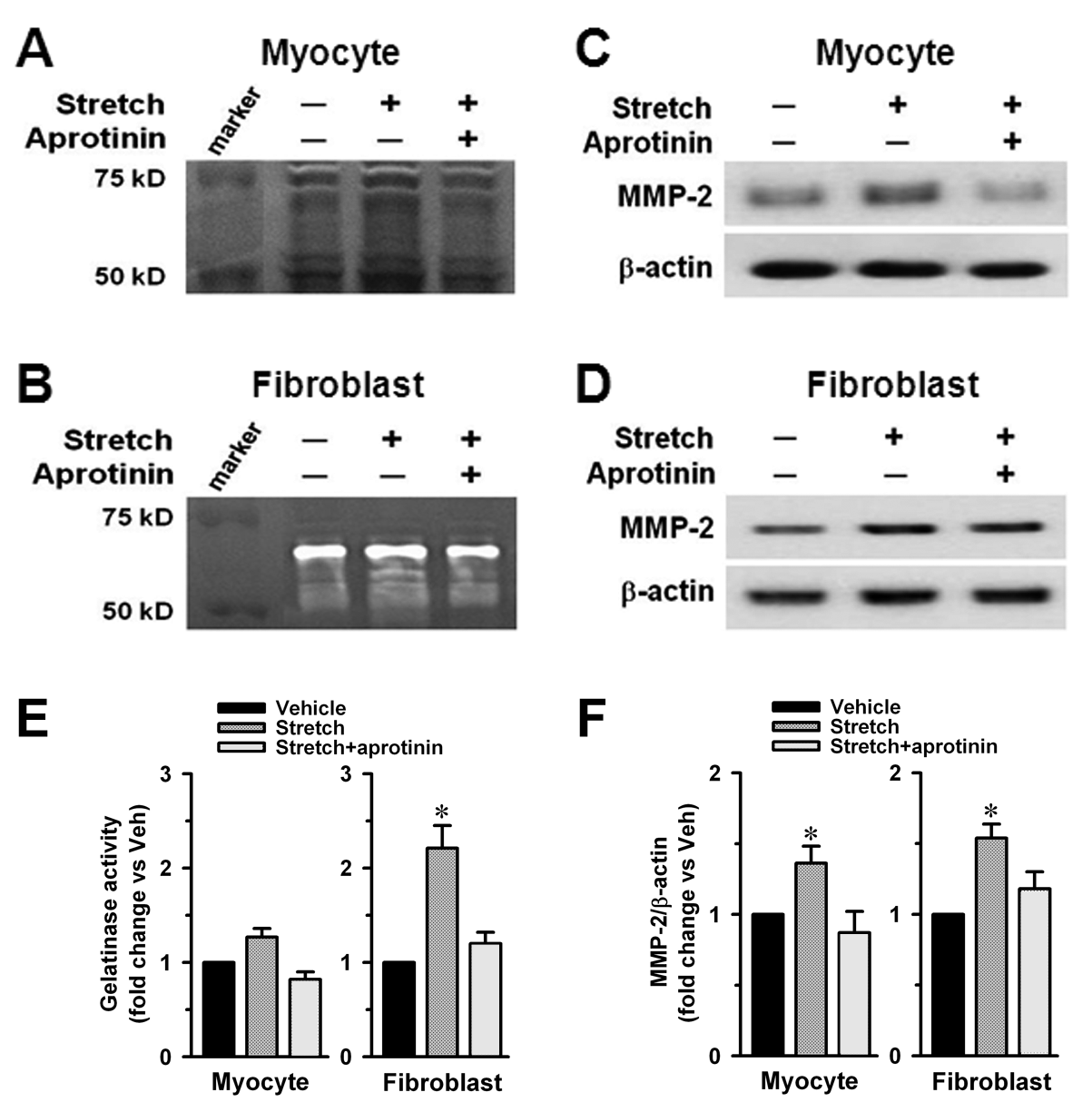

Supplement: Figure S3 — Gelatinase activity and protein expression in stretched cells. Gel zymography of gelatinase activity and MMP-2 protein expression in 24 hrs of cyclic stretch with or without aprotinin in adult rat cardiac myocytes and fibroblasts. There is increased MMP-9 activity at 78 kDa in cardiomyocytes (Panel A) and MMP-2 activity at 62 kDa in fibroblasts (Panel B) after 24 hrs of stretch. Panels C and D, MMP-2 protein expressions were increased in response to stretch in both cells. The gelatinase activity and MMP-2 protein expressions were both reduced by aprotinin treatment. Values are expressed as mean±SEM. n = 4–6 in each group. *P<0.05 vs. Vehicle. (TIF) [file pone.0040110.s003.tif]

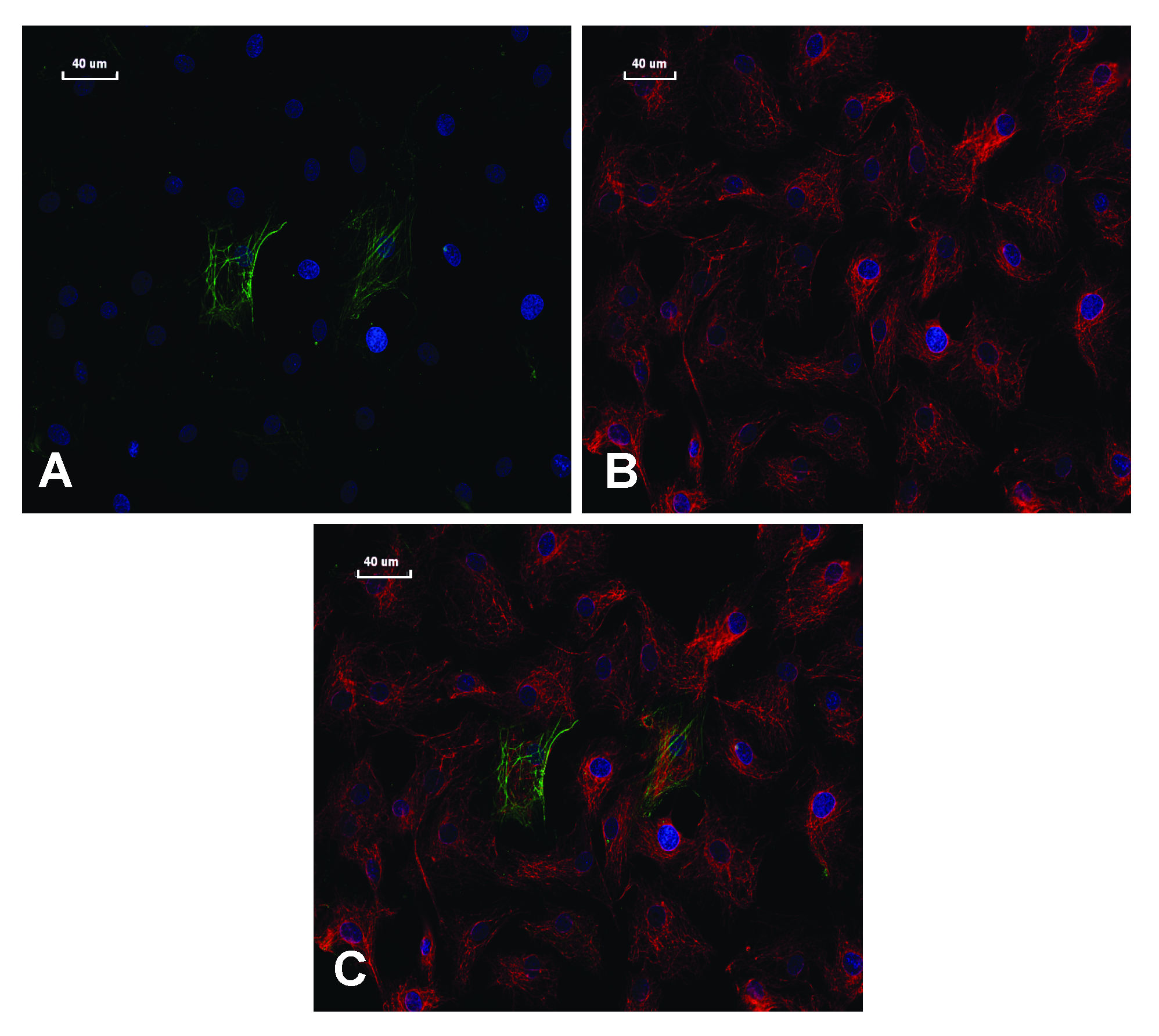

Supplement: Figure S4 — Immunofluorescence revealed phenotype of cardiac fibroblast. Cardiac fibroblasts used in our studies were at passage one. From the staining of the fibroblasts with alpha smooth muscle actin (A, green), vimentin (B, red) and DAPI (nuclei, blue), we showed >95% purity (C, composite) of the prep and little myofibroblast differentiation. (TIF) [file pone.0040110.s004.tif]
